# Upper Airway Alarmin Cytokine Expression in Asthma of Different Severities

**DOI:** 10.3390/jcm13133721

**Published:** 2024-06-26

**Authors:** Hazel Marriott, Marc Duchesne, Subhabrata Moitra, Isobel Okoye, Luke Gerla, Irvin Mayers, Jalal Moolji, Adil Adatia, Paige Lacy

**Affiliations:** Division of Pulmonary Medicine, Department of Medicine, University of Alberta, Edmonton, AB T6G 2R3, Canada; tjmarrio@ualberta.ca (H.M.); mduchesn@ualberta.ca (M.D.); moitra@ualberta.ca (S.M.); okoyei@mcmaster.ca (I.O.); lgerla@ualberta.ca (L.G.); imayers@ualberta.ca (I.M.); moolji@ualberta.ca (J.M.)

**Keywords:** TSLP, IL-25, IL-33, GINA, nasal, buccal, throat, epithelial cells

## Abstract

**Background:** The secretion of alarmin cytokines by epithelial cells, including thymic stromal lymphopoietin (TSLP), interleukin (IL)-25, and IL-33, initiates inflammatory cascades in asthma. However, alarmin cytokine expression in the upper airways in asthma remains largely unknown. **Methods:** We recruited 40 participants with asthma into four groups as per the Global Initiative for Asthma (GINA) steps (10 in each group of GINA 1/2, 3, 4, and 5). Cells were derived from nasal, buccal, and throat brushings. Intracellular cytokine expression (TSLP, IL-25, and IL-33) was assessed by flow cytometry in cytokeratin 8^+^ (Ck8^+^) epithelial cells immediately following collection. **Results:** TSLP was significantly increased (*p* < 0.001) in GINA 5 patients across nasal, buccal, and throat Ck8^+^ epithelial cells, while IL-25 was elevated in nasal and throat samples (*p* < 0.003), and IL-33 levels were variable, compared with GINA 1–4 patients. Individual GINA subgroup comparison showed that TSLP levels in nasal samples from GINA 5 patients were significantly (*p* = 0.03) elevated but did not differ between patients with and without nasal comorbidities. IL-25 and IL-33 (obtained from nasal, buccal, and throat samples) were not significantly different in individual groups. **Conclusions:** Our study demonstrates for the first time that Ck8^+^ nasal epithelial cells from GINA 5 asthma patients express elevated levels of TSLP.

## 1. Introduction

Asthma affects more than 300 million people globally, up to 10% of which are considered to fall under the diagnostic criteria for severe asthma [1,2]. Approximately half of severe asthma is poorly controlled, with a significant health burden and impact on quality of life despite adherence to standard inhaled therapies, including inhaled corticosteroids (ICSs) and long-acting β_2_ agonists (LABAs) [3]. The majority of these patients have evidence of type 2 (T2) inflammation and are candidates for biologic therapies directed against T2 inflammatory mediators (e.g., immunoglobulin E (IgE), interleukin (IL-4)/IL-13, IL-5, and thymic stromal lymphopoietin, (TSLP)); however, it remains a challenge to determine which therapeutic is likely to work for an individual patient [4,5].

Epithelial cell-derived alarmin cytokines, TSLP, IL-25, and IL-33, have recently emerged as playing important roles in the activation of inflammatory responses in the airways. These cytokines are released by airway epithelial cells in response to numerous biological and environmental triggers and initiate a cascade of downstream immune responses leading to airway inflammation in asthma patients [6,7]. TSLP is released in response to airborne triggers including allergens, pollutants, cigarette smoke, and viral infection [6,7,8,9,10,11,12]. As a master alarmin cytokine, TSLP plays a role in the activation of a range of immune cells, including dendritic cells, T-helper 2 cells, mast cells, eosinophils, and ILC2s [13,14,15,16,17,18]. The release of IL-25 is induced by exposure to common protease allergens, including house dust mites, leading to the induction of allergic inflammation through direct activation of eosinophils and T-helper 2 cells and resulting in increased release of IL-4, IL-5, IL-13, and airway eosinophilia [6,19,20,21]. IL-25 also contributes to structural changes in the airway by inducing collagen secretion by and the proliferation of fibroblasts, contributing to airway remodeling and hyper-responsiveness in asthma [22,23]. IL-33 is expressed in a wide variety of tissue cells, including epithelial and endothelial cells, and is released into the airways after cellular damage or following exposure to inhaled allergens; it synergistically promotes the activation and recruitment of type 2 innate lymphoid cells (ILC2s) along with TSLP [24,25].

Molecular signatures in nasal, buccal, and throat samples have been previously explored as potential surrogates for asthma, with varying degrees of success. Previously, buccal brushings have been shown to have detectable changes in both epigenetics and gene expression when comparing subjects with or without asthma [26,27,28]. To our knowledge, throat epithelial cells have not been directly studied in the context of asthma, although a recent study has identified the throat microbiota as a potential biomarker for asthma exacerbations despite continued ICS therapy [29]. The nasal epithelium has been more robustly studied in the context of asthma, with the nasal microbiome, nasal lavage, and nasal brushings all showing potential as asthma biomarkers [30,31,32,33]. It has previously been suggested that nasal epithelial cells isolated from asthmatic patients and cultured ex vivo express greater levels of TSLP following rhinovirus infection as compared to cells from healthy individuals [12]. However, despite these recent advances, studies measuring the in situ expression of alarmin cytokines in nasal, buccal, and throat epithelia in asthma are lacking. We sought to assess whether the upper airway epithelium of patients with asthma may be an alternative source for the measurement of intracellular alarmin cytokines (TSLP, IL-25, and IL-33) in asthma and whether their expression is altered in association with asthma severity.

## 2. Materials and Methods

### 2.1. Study Design and Participants

In this cross-sectional study, we recruited 40 adult (>18 years) participants with asthma who received maintenance therapy (ICS, LABA, etc.) for their symptoms but were naïve to biologic therapy. Asthma was diagnosed and categorized by severity according to the Global Initiatives for Asthma, with 10 participants from each GINA category (GINA 1 and 2 were merged due to overlapping medication levels) [1]. Demographic information, medication use, comorbidities, and emergency department visits in the past 12 months were collected through review of the patients’ medical records. None of the patients experienced exacerbations within the previous 7 days before sampling. All patients were on stable maintenance therapy for ≥3 months before sample collection. This study was approved by the Health Research Ethics Board of the University of Alberta (Pro00106537), and all participants provided written informed consent.

### 2.2. Sample Collection and Processing

Participants in this study were requested to rinse their sinuses using a prewarmed bottle of NeilMed Sinuflow ReadyRinse^®^ solution (400 mL, NeilMed^®^ Pharmaceuticals, Santa Rosa, CA, USA) and to rinse out their mouths and throats with sterile 0.9% saline solution (50 mL) prior to sample collection. Samples were then collected from the nasal, buccal, and throat cavities of participants using a cytology brush (Pap-Pak^®^ Cytosoft™ Medical Packaging Corporation, Camarillo, CA, USA). After collection of cells, cytology brushes were immediately inserted into 15 mL conical tubes containing 10 mL complete bronchial epithelial growth media (BEGM™ Lonza, Basel, Switzerland). To detach cells from brushes, tubes were briefly vortexed on a lower speed before centrifugation at 300× *g* for 10 min at 4 °C. Cytobrushes were removed, and cells were once again pelleted by centrifugation to account for any dislodging of cells during cytobrush removal (300× *g* for 10 min at 4 °C). Supernatants were aspirated and the pellets were resuspended in 1 mL FACS buffer (0.5% BSA and 0.05% sodium azide in PBS, pH 7.4) and a 10 μL aliquot was taken for counting using Trypan blue and an automated cell counter, Countessa 3 (Invitrogen, Waltham, MA, USA). 

### 2.3. Flow Cytometry

**Figure 1:**
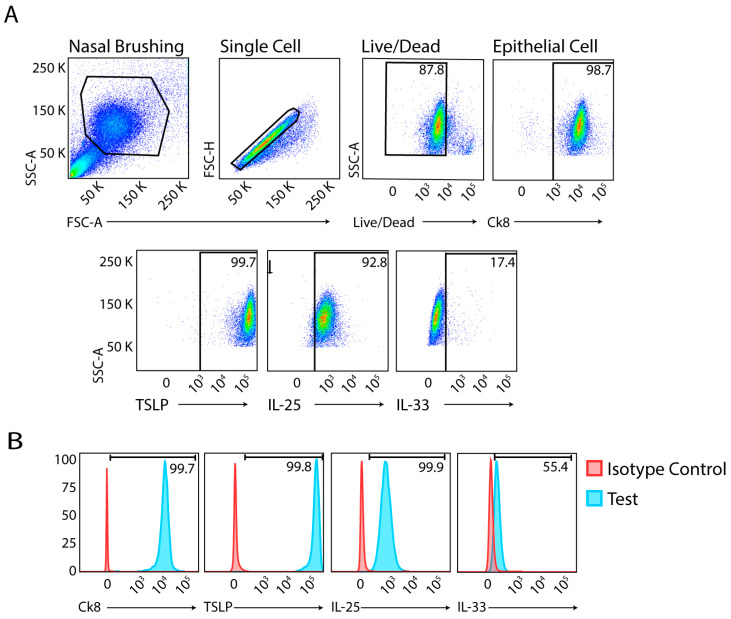
Flow cytometric analysis of alarmin cytokines in upper airway brushings. (**A**) Sequential gating strategy. **Top row**: representative nasal brushing sample, single cell selection, live/dead cell gating, and gating for epithelial cells based on Ck8 labelling. **Bottom row**: Individual alarmin cytokine labelling of epithelial cells. (**B**) Histograms showing isotype controls and test antibodies for Ck8, TSLP, IL-25, and IL-33 on appropriately gated epithelial cells.

Cells were briefly washed in FACS buffer prior to staining to remove excess debris and mucus. A LIVE/Dead™ Fixable Aqua—Dead Cell Stain Kit (Life Technologies, Burlington, ON, Canada) was used for dead cell discrimination prior to fixation and permeabilization of cells. Live/dead staining was performed for 25 min at 4 °C. Cells were then fixed and permeabilized using a BD Cytofix/Cytoperm™ fixation/permeabilization kit (BD Biosciences, Mississauga, ON, Canada), according to the manufacturer’s instructions. After blocking, cells were labelled with antibodies to cytokeratin 8 (Ck8) to identify epithelial cells and alarmin cytokines to quantify their expression levels according to GINA steps (Figure 1). 

Primary antibodies were used for flow cytometry as follows: anti-TSLP antibody (rabbit polyclonal antibody, ABT330, Millipore Sigma Mississauga, ON, Canada), Ck8 conjugated to Alexa Fluor 647 (EP1628Y, Abcam, Waltham, MA, USA), anti-IL-25 antibody (182203, R&D Systems, Minneapolis, MN, USA), and IL-33 conjugated to phycoerythrin (PE, 40015C, R&D Systems). To detect TSLP labelling, cells were incubated with Alexa Fluor 488 F(ab’)_2_ fragment of goat anti-rabbit IgG (H+L) (A11070, Life Technologies Corporation, Eugene, OR, USA). IL-25 labelling was detected with PE-Cy7-conjugated rat anti-mouse IgG (M1-14D12, eBioscience, ThermoFisher Scientific, Mississauga, ON, Canada). Primary and secondary antibodies were each incubated for 30 min at 4 °C. Isotype control antibodies were used as follows: Alexa Fluor 647 mouse IgG_1_κ Isotype Control (MA5-18167, BD Biosciences), mouse IgG_1_ FITC-conjugated negative control antibody (400109, Bio-Rad, Mississauga, ON, Canada), and PE-conjugated goat IgG isotype control (IC108P, R&D Systems). Fluorescence Minus One (FMO) controls were also used to identify and gate positive populations. Immunolabelled cells were acquired on an LSR Fortessa-SORP (BD Biosciences) and analyzed using FlowJo software (version 10, Ashland, OR, USA). 

### 2.4. Statistical Analysis

Descriptive statistics are presented as mean (standard deviation [SD]), median (interquartile range [IQR]), and frequency (%) for continuous, count, and categorical variables, respectively. We used multivariable quantile regression to assess the differences in each cytokine across the GINA subgroups. We tested age, sex, smoking status, and nasal comorbidities as potential confounders; however, we retained only age and sex in the models based on Akaike’s information criterion [34]. We performed exploratory stratification analyses to test the differences in cytokine expression among patients with and without nasal comorbidities (allergic rhinitis and chronic rhinosinusitis with/without nasal polyps). All analyses were performed with a complete case approach using STATA 18 (StataCorp, College Station, TX, USA) and *p*-values < 0.05 were considered statistically significant.

## 3. Results

Of all participants, 63% were female, the mean (SD) age was 41 (16) years, and 85% were non-smokers (Table 1). A total of 36 (90%) were on ICS (as daily maintenance therapy or as needed with a reliever) and 13 (33%) had one or more nasal comorbidities. Six patients without and six with a documented diagnosis of nasal comorbidities were receiving treatment with intranasal corticosteroids. 

We processed nasal, buccal, and throat samples immediately (within 1 h) after collection from participants and subjected these to flow cytometry analysis without stimulation. After gating for live cells and singlets, and using Ck8 as the epithelial cell marker, we found detectable levels of alarmin cytokines in all samples obtained from patients in GINA steps 1–5 (Figure 1). 

The median (IQR) MFIs of TSLP, IL-25, and IL-33 were 6590 (3829–19,032), 3936 (3104–8061), and 923 (548–2010), respectively, for all Ck8^+^ cells in nasal samples across GINA steps. In buccal samples, the median (IQR) MFIs of TSLP, IL-25, and IL-33 were 7766 (3578–15,120), 14,635 (3702–23,095), and 1065 (438–3566), respectively, and in throat samples, the MFIs were 6615 (3281–13,712), 8109 (4251–13,795), and 1115 (649–3113), respectively (Table 1).

**Figure 2:**
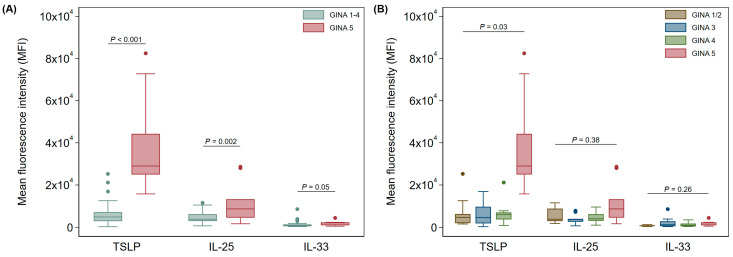
Expression of alarmin cytokines in nasal Ck8^+^ epithelial samples (**A**) between GINA 1–4 and GINA 5 and (**B**) across all GINA groups. Data are shown as median (IQR) (box and whisker) and 95% confidence intervals (error bars) unless otherwise stated, and *p*-values were obtained from quantile regression models adjusted for age and sex.

In nasal brushings, GINA 5 asthma patients expressed significantly higher levels of both TSLP and IL-25 when compared to all other asthma patients combined (GINA 1–4, *p* < 0.001 and *p* < 0.002, respectively, after adjusting for age and sex, Figure 2A). There was no significant change in IL-33 expression between the two groups (Figure 2A). 

When GINA groups were broken down to 1/2, 3, 4, and 5, there was still a significant elevation in TSLP expression in nasal epithelial cells from GINA 5 patients; however, significance was lost for IL-25 and IL-33 (Figure 2B). Further, there was no significant difference in nasal cytokine expression for any comparison of GINA 1 through 4 patients after adjusting for age and sex (Figure 2B). 

**Figure 3:**
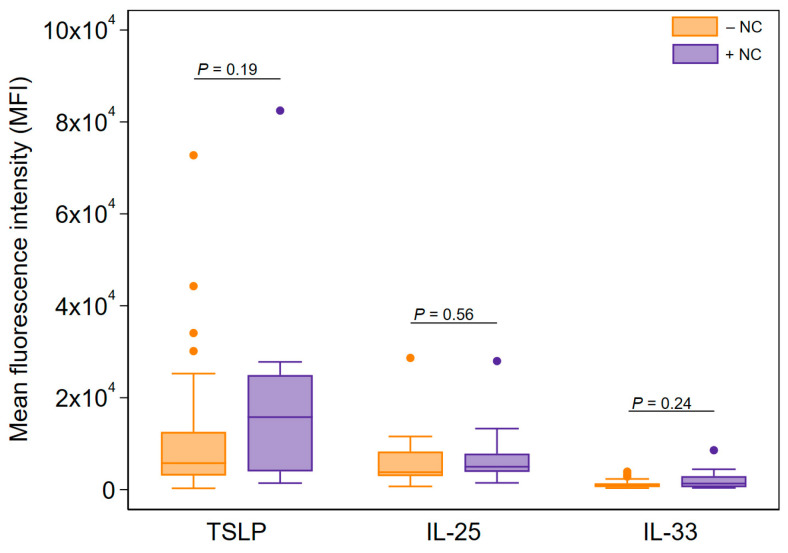
Stratification analysis of alarmin cytokine expression in nasal Ck8^+^ epithelial samples among all asthma patients stratified by nasal comorbidities. *p*-values were obtained from quantile regression adjusted for age and sex. −NC: no comorbidities; +NC: with comorbidities.

In an exploratory stratification analysis, we found a three-fold increase in Ck8^+^ epithelial cell TSLP expression in nasal brushings from participants with recorded nasal comorbidities (median MFI: 15,774) compared with those lacking nasal comorbidities (median MFI: 5759), although this was not statistically significant (Figure 3). The expression of IL-25 and IL-33 was also elevated in Ck8^+^ epithelial cells from participants with nasal comorbidities; however, none of the measurements differed significantly between patients with or without nasal comorbidities in terms of the threshold for statistical significance after adjusting for age and sex (Figure 3). 

**Figure 4:**
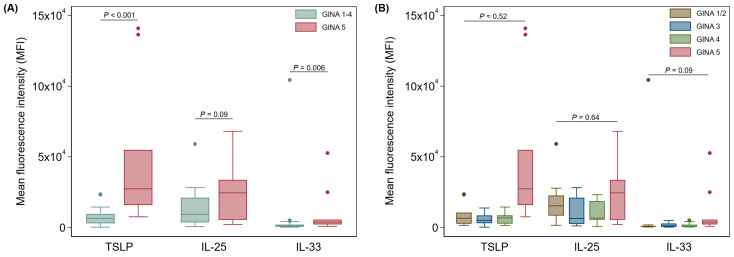
Expression profile of alarmin cytokines in buccal Ck8^+^ epithelial samples (**A**) between GINA 1–4 and GINA 5 and (**B**) across all GINA groups. Data are shown as median (IQR) (box and whisker) and 95% confidence intervals (error bars) unless otherwise stated, and *p*-values were obtained from quantile regression models adjusted for age and sex.

In buccal Ck8^+^ epithelial cells in patient samples, there was a significant elevation in TSLP in GINA 5 asthma patients when compared to all other patients (*p* < 0.001 after adjusting for age and sex, Figure 4A). Levels of IL-25 did not show a significant difference in GINA 1–4 compared with GINA 5. IL-33 showed significant differences between the GINA 1–4 and GINA 5 groups, but levels were highly variable for this cytokine. When each GINA stratification was compared to all others, we observed no significant changes in TSLP, IL-25, or IL-33 after adjustment for age and sex in buccal samples (Figure 4B). 

**Figure 5:**
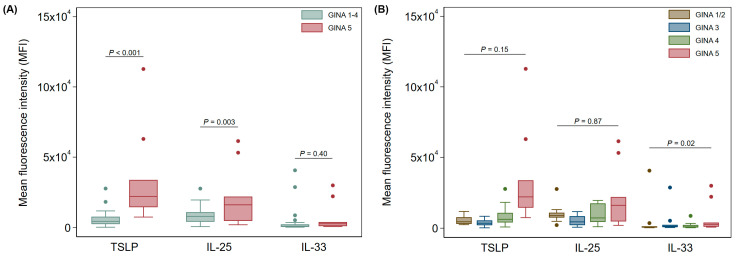
Expression profile of alarmin cytokines in throat Ck8^+^ epithelial samples (**A**) between GINA 1–4 and GINA 5 and (**B**) across all GINA groups. Data are shown as median (IQR) (box and whisker) and 95% confidence intervals (error bars), and *p*-values were obtained from quantile regression models adjusted for age and sex.

A similar pattern was observed in the expression profiles of throat Ck8^+^ epithelial cells in patient samples, with significant increases in TSLP and IL-25 when comparing GINA 5 to all other asthma patients (*p* < 0.001 and *p* = 0.003, respectively, after adjusting for age and sex, Figure 5A). IL-33 showed no significant difference in throat samples between GINA 1–4 and GINA 5 patients. Once again, when each GINA group was compared by stratification, we found no significant changes in TSLP, IL-25, and IL-33 across the GINA groups (Figure 5B).

## 4. Discussion

In this study, we implemented a novel, non-invasive, and rapid method of measuring alarmin cytokine expression in the upper airways of asthma patients. This flow cytometry-based technique enabled clear detection of TSLP in all samples, and we found significant elevations of TSLP in nasal, buccal, and throat Ck8^+^ epithelial cells from patients in the GINA 5 group when compared with those in GINA 1–4. In addition, we observed significant increases in IL-25 expression in the nasal and throat epithelia of GINA 5 patients in comparison to GINA 1–4. Finally, while we saw significant changes in IL-33 in GINA 5 patients, these were highly variable and generally inconsistent between groups in all samples. 

In contrast, when we stratified the GINA groups and compared the levels of alarmin cytokines in all samples, we found that there were no differences among the GINA 1/2, 3, and 4 subgroups of patients with asthma for TSLP, IL-25, and IL-33. This finding suggests that these samples do not reflect disease severity in these categories of patients.

To our knowledge, this is the first study directly comparing the nasal, buccal, and throat epithelia of different severities of asthma, with previous work focusing on broader comparisons between healthy and asthmatic subjects [26,27,28,29]. Furthermore, no significant changes were observed in IL-33 levels across all GINA categories, which agrees with a recent study on TSLP and IL-33 serum levels in mild asthma patients, showing a slight reduction in sera in mild asthma compared to that in healthy controls [35]. Increased TSLP expression in nasal epithelial cells among those with nasal comorbidities, although not significant, is also consistent with previous studies in allergic rhinitis and chronic rhinosinusitis [36,37].

In contrast to studies showing elevated alarmin cytokine expression in the bronchial epithelium in correlation with increasing severity of asthma, we observed no differences in cytokine expression among GINA 1–4 subgroups in nasal, buccal, and throat epithelial samples. This observed lack of difference is most likely explained by distinct cell types present in nasal, buccal, throat, and lung epithelia [38,39]. Furthermore, it has previously been shown that nasal epithelial cells have distinct cytokine responses from bronchial epithelial cells in vitro [40,41]. Additional work is needed to fully appreciate the differences in cytokine regulation between the upper and lower airway epithelia. 

Our finding of high nasal, buccal, and throat expression of alarmin cytokine expression in GINA 5 patients may help to guide therapy for patients with severe asthma. A key feature of severe asthma is persistent, corticosteroid-insensitive airway inflammation. Corticosteroid resistance is thought to be mediated by abundant type 2 cytokine production by group 2 innate lymphoid cells (ILC2s) [42], and is dependent on TSLP [43]. Currently, asthma severity is determined post hoc based on the intensity of the required treatment, which can delay necessary therapy in severe disease. The application of upper airway TSLP and IL-25 measurement could therefore provide guidance on appropriate therapy in severe asthma; however, future studies are required to explore this possibility.

The upstream position of alarmin cytokines as orchestrators of allergic airway inflammation has been harnessed as a strategy for asthma therapy [44,45,46]. The anti-TSLP monoclonal antibody, tezepelumab, has been shown to improve exacerbation rates, lung function, symptom control, and quality of life in patients with severe asthma [47,48]. However, severe asthma phenotypes are highly heterogeneous due to inherent differences in etiopathological mechanisms, and thus it may be valuable to measure the expression of inflammatory mediators prospectively in severe asthma patients to determine which patients are most likely to benefit from this drug. While bronchial brushings and induced sputum may be used for measuring cytokine levels, harvesting cells through these methods is technically challenging, invasive, and not always feasible, particularly in patients with reduced lung function or scant sputum production [5]. Therefore, the upper airways may be an attractive target for study as potential surrogates of the lung. 

While this is the first report demonstrating increased upper airway expression of TSLP in severe asthma patients, there are important study limitations. This study was cross-sectional in design and could not provide insight into the effects of asthma exacerbations or treatment on the expression of cytokines. The number of patients within each GINA severity group was small, and GINA severity groups were determined by prescribing physicians, which may not fully reflect disease severity. An analysis of alarmin expression within clinically relevant subgroups such as GINA 5 patients with and without nasal polyposis was thus not possible due to the small sample size. Due to limited sample sizes, a comparison between patients with nasal comorbidities by GINA steps was similarly not possible. Additionally, patients with nasal comorbidities were identified post hoc through the review of patient records. It cannot be excluded that some patients’ diagnoses were not appropriately documented. We also did not include a medication washout period prior to sample collection. The issue of mouth/throat deposition of inhaled medications is well documented; deposition of ICS may have suppressed the endogenous alterations in alarmin cytokine expression in these samples [49,50,51,52]. Similarly, in nasal samples the effect of inhaled nasal steroids on alarmin cytokine expression cannot be excluded. Although nasal epithelial TSLP may be effective at identifying patients with severe or steroid refractory asthma, it may be a poor biomarker in mild-to-moderate disease. Compliance with treatment was determined by patient reports, so the severity of disease in noncompliant patients may have been misidentified. We speculate that nasal sampling could be used to identify patients with more severe disease; however, this requires a future prospective study rather than the current cross-sectional study. Additionally, we could not assess TSLP in patients before and after the initiation of inhaled steroids. Larger samples sizes are required to fully understand the interplay of these diseases on the expression of alarmin cytokines in the upper airway epithelium. 

## 5. Conclusions

In conclusion, upper airway alarmin cytokine expression can be measured using a novel flow cytometric assay on nasal brushings. The expression of TSLP and, to a lesser extent, IL-25 is increased in patients with severe asthma. The utility of upper airway TSLP as a biomarker of severe asthma and to guide therapy warrants further study. 

## Figures and Tables

**Table 1:** Participant demographics and clinical characteristics.

	All Patients (N = 40)
Demographics	
Age, yr, mean (SD)	40.9 (15.8)
Female, n (%)	24 (60.0)
Smoking history, n (%)	
Never smoker	35 (87.5)
Clinical Characteristics	
GINA step, n (%)	
GINA 1/2	10 (25.0)
GINA 3	10 (25.0)
GINA 4	10 (25.0)
GINA 5	10 (25.0)
Nasal comorbidities, n (%)	13 (33.0)
Nasal polyps, n (%)	4 (10.0)
Medications used, n (%)	
ICS/LABA maintenance	31 (77.5)
SABA reliever	17 (42.5)
Nasal steroid spray	12 (30.0)
Anti-allergic oral drugs *	11 (27.5)
Oral corticosteroid maintenance	1 (2.5)
At least one exacerbation, past 12 months, n (%)	4 (10.0)
Laboratory Investigations	
Nasal samples	
TSLP (MFI), median (IQR)	6590 (3829–19,032)
IL-25 (MFI), median (IQR)	3936 (3104–8061)
IL-33 (MFI), median (IQR)	923 (548–2010)
Buccal samples	
TSLP (MFI), median (IQR)	7766 (3578–15,120)
IL-25 (MFI), median (IQR)	14,635 (3702–23,095)
IL-33 (MFI), median (IQR)	1065 (438–3566)
Throat samples	
TSLP (MFI), median (IQR)	6615 (3281–13,712)
IL-25 (MFI), median (IQR)	8109 (4251–13,795)
IL-33 (MFI), median (IQR)	1115 (649–3113)

*: anti-allergic drugs include antihistamines and leukotriene modifiers.

## Data Availability

Data are contained within the article and the dataset is available on request from the authors.
